# An investigation into prospective memory in children with developmental dyslexia

**DOI:** 10.3389/fpsyg.2014.01308

**Published:** 2014-12-04

**Authors:** Azizuddin Khan

**Affiliations:** ^1^Psychophysiology Laboratory, Department of Humanities and Social Sciences, Indian Institute of Technology BombayMumbai, India; ^2^Facultad de Psicología, Psicología Educativa, Universidad de CuencaCuenca, Ecuador

**Keywords:** attention, memory, meta-memory, questionnaire, and reading acquisition

## Abstract

Developmental dyslexia hinders reading and writing acquisition of around 5–10% of the children all over the world. However, little is known about role of prospective memory among dyslexics. Prospective memory is realization of delayed intention. Realization of delayed intention requires self initiated process. The present study explored the role of memory (prospective and retrospective memory), meta-memory and attention among dyslexic's children. One hundred and fifteen children (51 dyslexics and 64 normal controls) participated in the study. Prospective and retrospective memory questionnaire, everyday attention questionnaire and meta-memory were administered on children. Analysis of variance was used to analyses the data. All the main effects were significant. Some interactions were also found to be significant. Results suggest that dyslexic's performance on memory (prospective and retrospective memory) was worse than normal control. Meta-memory influences both dyslexics and normal control on prospective and retrospective memory. However, meta-memory affected dyslexics much more than normal control group. Similarly, significant differential effects were observed for simple, difficult and mixed attentional condition among between dyslexics and normal control. Dyslexic's performance was deteriorated as compared to normal control group. The findings of the study are discussed in the light of the existing literature.

## Introduction

Developmental Dyslexia is a specific reading and writing disability despite of normal intelligence, educational instruction and socio-cultural opportunity (Dilling et al., 1991). It is expected that 5–10% of school going children are afflicted by developmental dyslexia (Shaywitz, 1998). Despite the great works have been done to understand the causes of dyslexia since the last 100 years, there is still controversy regarding the exact precursor and specific effect of the disorder. One of the potent and plausible hypotheses states that phonological deficit of verbal short term memory is one of the cause of the developmental dyslexia. The phonological loop evolved to facilitate the acquisition of language (Baddeley et al., 1998). The segmentation of text into graphemes is considered one of the indicators of acquisition of reading ability. Its capacity is a good predictor and indicator of the ability of children and adults to learn new language. (Baddeley, 2003). Dyslexics have problem in extracting phonemes from the text. Research has indicated that the conversion of the written word image into its phonological equivalent in the brain is crucial in the normal process of reading fluently. Failure to develop an association between letter and sound is a major cause of reading and spelling impairment in many or most instances of developmental dyslexia (Ramus, 2004).

The bulk of research in the past decade has investigated an approach of the phonological deficit hypothesis in dyslexics. Phonological awareness enables the listener to recognize, identify, and manipulate basic language sounds (phoneme segmentation, blending, and deletion). Babies begin to develop phonological awareness as soon as they absorb and acquire the sounds of their native language (Buckley, 2003). The child hears the sounds of words and attempts to repeat them as heard. According to the phonological deficit hypothesis, dyslexics have a difficult time with written language because they have an impaired ability to deconstruct written words into phonemes and therefore it prevents word identification (Baddeley et al., 1998). Several studies since early 1990s have demonstrated that dyslexic children are deficient in dividing specific words into phonemes. For example if dyslexic children is asked to say “rock” without the “r” sound, they have greater difficulty with this phoneme deletion task than non-dyslexics.

Deficits in phonological processing are seen in the majority of children with dyslexia. The phonological deficit theory of dyslexia has received support from studies of dyslexia across the life span. A substantial number of studies of adults with a developmental history of dyslexia have reported persisting deficits in phonological awareness and in phonological processing tasks (Felton et al., 1990; Pennington et al., 1990; Bruck, 1992; Snowling et al., 1997). It affects the acquisition of phonic skills in reading and spelling so that unfamiliar words are frequently misread. The acquisition of the phonological system of one's native language proceeds via consolidation of short term acoustic and ensuing phonological representations into the long term memory codes (Naatanen and Winkler, 1999). Sensory memory can be understood into three phases (Naatanen, 1990). The incoming information from the environment first encoded into a neuro-physiological mechanism of short term memory for very short period of time that last about 200–300 ms. If the information is attended it goes to second phase of sensory memory and last about 10–20 s. Information at this phase is available for the conscious perception and for selective attention. It processes information in top down fashion. It also provides basis for sound perception as an auditory event. Lastly, the long term sensory memory resides in the brain for longer period and sometime whole life. Consequently, auditory sensory memory becomes crucial in the development of short term memory/WM for speech, which is involved in language learning. Role of working memory is well established.

However, there is not a single study to my knowledge investigating prospective memory performance in children with dyslexia. Prospective memory is memory for action to be performed in the future such as remembering to take medicine after every 2 h (Einstein and McDaniel, 1990). Aristotle labeled an action to be performed in future as “memory for the future” (Herrmann and Chaffin, 1988). Unlike prospective memory, retrospective memory involves remembering of past information such as recalling the name of a person whom one had met a few days back (Crawford et al., 2006). The concept of prospective memory can be better understood by comparing and contrasting it with retrospective memory. Prospective memory is characterized by future orientation and intention. It passes through various phases and involves planning and monitoring of information regarding the to-be-performed action, whereas retrospective memory is past oriented and incidental. According to psychologists, prospective memory is the memory for intent, whereas retrospective memory is the memory for content (Kvavilashvili, 1987; Marsh et al., 1998; McDaniel and Einstein, 2007). In prospective memory there is no obvious and external cue that prompts a person to retrieve the information. However, in retrospective memory there is an external prompting for remembering. Studies have shown divergent views with respect to the relationship between retrospective and prospective memory (Meacham, 1988). Some studies have clearly indicated that there is no correlation between retrospective and prospective memory (Kvavilashvili, 1987; Einstein and McDaniel, 1990; Maylor, 1990), while others have demonstrated that a positive correlation exists between the two (Huppert et al., 2000).

Prospective memory involves four phases for realization of delayed intention and action associated with it (Brandimonte and Passolunghi, 1994).

Phase 1 is concerned with the encoding of the content of to-be-remembered information and involves (i) formation of intention, (ii) retrospective component, and (iii) prospective component. Phase 2 refers to the performance delay where intended action should be retrieved for successful performance of prospective memory. Phase 3 involves initiation and execution of intended action, and phase 4 involves the evaluation of the outcome which relates to remembering having performed the action, so as not to repeat it (cancelation of intention stage).

According to Einstein and McDaniel (1990), there are two components of any prospective memory task. The first is a retrospective memory component which deals with the retention of the action and “target event” or retrieval context. The second component is characterized by a prospective memory component that requires retrieval of action at an appropriate time or in response to an appropriate event. These two components correspond to delay and performance interval. Phase 1 refers to retrospective component and phases 2–4 deal with the prospective component.

Though successful prospective memory requires remembering to remember, all prospective memory tasks have a retrospective memory component so that a “pure” prospective memory task may not exist (Maylor, 1993). The retrospective component of prospective memory consists of retention of the action and retrieval context and consists of the action (what), intent (that), and retrieval context (when). The retrospective component thus involves only formation and encoding of intention and action (phase 1). The prospective component of prospective memory, on the other hand, refers to the retrieval of the action “at the appropriate time or in response to the appropriate event” (Einstein and McDaniel, 1990, 2005). It includes retention interval, performance interval, initiation and execution of intended action and evaluation of outcome (phases 2–4) (Brandimonte, 1995; Brandimonte et al., 2001).

Prospective memory requires two different things to be remembered: “What is to be done?” and “at what time, or in which situation the action should be performed?” Thus, prospective memory performance may be external cue based or subject monitored. Research has demonstrated that two kinds of prospective memory exist: time-based and event-based (Einstein et al., 1995).

Time-based prospective memory task requires that the subject remembers to perform some action at a certain time or after a certain period of time has elapsed (e.g., remembering to attend a meeting at 11 a.m.). There is no explicit or specific external event which works as a cue for the to-be-performed action. People must remember on their own to monitor the passage of time and initiate action. Event-based prospective tasks are those in which the intended action is to be performed when a certain external event occurs (e.g., to give a message to someone on seeing that person (Smith et al., 2010).

Prospective memory involves working memory which is also instrumental in language learning (Ackerman and Dykman, 1993; Marsh and Hicks, 1998; Brunswick et al., 1999; Wang et al., 2013). Working memory has three components- phonological loop, visuosa-patial sketchpad and central executive (Baddeley et al., 1998). Dyslexic showed poor performance involving material that can be coded with verbal form, but there was no group difference when non-verbal material was used (Vellutino et al., 1975; Liberman et al., 1982). Since verbal material can give rise to the activation of phonological codes, it is possible that verbal memory difficulty may emanate from inefficient use of phonological codes. Poor readers show phonological deficit (Liberman and Shankweiler, 1985; Mody et al., 1997). It has also been observed that Further successful prospective memory requires planning, monitoring and self-initiation. Dyslexic may show poor performance on prospective memory as compared to retrospective memory.

Similarly, memory about memory may also affect performance of dyslexic children. Belief about memory is quite critical among dyslexic children.

Meta-memory is a general term that refers to knowledge, beliefs, and feelings about memory (Dixon, 1989). It refers to knowledge and awareness of memory and was considered as accurate knowledge or truth about memory (Flavell, 1971). However, contemporary view of metamemory does not equate memory knowledge with accuracy or truth. Rather, metamemory is treated like other mental representations which includes accurate and naïve representation of memory. Moreover, both accurate and naïve beliefs influence memory behavior and performance (O'Sullivan, 1993). Memory self-efficacy is a dimension of meta-memory which deals with the attitudes and reflects one's own memory ability (Hultsch et al., 1988). Meta-memory influenced both retrospective and prospective memory. However, since it is memory self-efficacy, it affects prospective memory most as compared to retrospective memory. Studies on retrospective memory have clearly shown that metamemory does affect memory. But the relationship between metamemory and prospective memory is still not very clear.

Thus, the experiment was designed with the following specific objectives. The first objective was to compare prospective and retrospective memory performance between dyslexic and non-dyslexic children. The second aim was to investigate belief about memory among dyslexic and non-dyslexic children. Finally, role of attention in dyslexics and non-dyslexics children was assessed.

## Methods

### Subjects

One hundred and fifteen subjects participated in the experiment. They were class 5th to class 12th students with the mean age of 12.23, *SD* = 1.29. Out of 115, 51 subjects were diagnosed as learning disabled. 64 subjects were without any disability and drawn from Mumbai suburban schools. Written consents were obtained from parents of children participated before beginning of the study.

### Stimulus material

To obtain personal information of the subject, to present the objects on a computer screen one after another in a controlled manner and to record the response of the subject, a computer program using visual basic was developed.

Three questionnaires were utilized. The first was Prospective and Retrospective Memory Questinnaire (PRMQ) developed by Smith et al. (2000). It consists of 16 self-report items and taps minor memory mistakes that everyone makes from time to time. Eight items measure prospective memory and 8 retrospective memories. The PMRQ also measures self-cued memory as well as environmentally-cued memory (both with short- and long-term). Participants were requested to tick on the appropriate place how often each of these things happen to them on 5-point scale: Very Often, Quite Often, Sometimes, Rarely, and Never. Ratings were assigned numerical values of 5 (very often) to 1(never).

Everyday Memory Questionnaire (EMQ) was developed by Sunderland et al. (1984) consists of 37 items which measures self-reported meta-memory. Participants were asked to answer each item on a 5-point scale: Very Poor, Poor, Do Not Know, Good, and Very Good. Ratings were assigned numerical values from 1 (Very Poor) to 5 (Very Good) respectively. High score on meta-memory indicates that individual feels more competent in memory situation. On the basis of meta-memory score, participants were divided into two groups: low meta-memory and high meta-memory participants.

The third questinnaire was “Everyday Attention Questionnaire (Martin, 1986). It consists of 18 items which measures to assess how easy or difficult people find it to pay attention in different everyday activities (e.g., “Imagine that you are carrying out some task you find easy such as peeling potatoes or knitting.” “I can always manage to solve difficult problems if I try hard enough,” “I can usually handle whatever comes my way”). Participants were asked to answer each item on 4-point scale: Not at all true, Hardly true, Moderately true, Exactly true. Ratings were assignes numerical values of 1 (very distracting) to 4 (very helpful). Subjects were asked to be as accurate as possible in giving the responses. Subjects were told that the information given by them will be confidential.

The Prospective and Retrospective Memory Questionnaire (PRMQ) was administered after the completion of EMQ. Later on Everyday Attention Questionnaire (EAQ) was administered.

### Design

The study used a 2(Memory: Prospective vs. Retrospective) × 2 (Term: Long term vs. Short term) × 2 (Cue: Self-cued vs. Environmental cue) × 2 (Group: Dyslexic vs. Normal control) x Meta-memory (Low vs. High) within factorial design, except last two factors, Group: Dyslexic vs. Normal control) and Meta-memory (Low vs. High) as between- subjects factors. The dependent measure was errors in prospective and retrospective memories in dyslexic and non-dyslexic group.

### Procedure

First, Wechsler Intelligence Scale for Children (WISC) -IV test, Woodcock–Johnson Tests of Cognitive Abilities, and Behavior Checklist for Screening for Learning Disability were employed for the screening of dyslexic's population.

After screening of dyslexics and control group, prospective and retrospective memory questionnaire was administered followed by meta-memory. Higher score in meta-memory indicates that individual feels more competent in memory situations. On the basis of meta-memory score, participants were divided into two groups- low-meta-memory and high meta-memory participants. The division of meta-memory into low and high was done on the basis of mean. Those who were above the mean were assigned as high on meta-memory while those who were below were assigned as low on meta-memory. Finally, everyday attention questionnaire was administered. Participants were informed that there were no right and wrong answers. However, they were asked to be as accurate as in responding. They were assured the confidentiality of their identity and were informed that their responses would be used only for research purposes.

## Results

Unless otherwise stated, only effects significant beyond at or 0.05 level are described. Memory (prospective vs. retrospective), EMQ (low vs. high) as between subjects and EAQ (low vs. high) were utilized as within subject factors.

The reliability of the data from all the three scales was found to be high. Cronbach's alpha was 0.75 for the prospective and retrospective memory, 0.73 for the meta-memory, and 0.74 for the everyday attention questionnaire. The mean and standard deviation scores for each question type are shown in Table 1.

**Table 1:** **Means and (Standard Deviation) memory error frequency rating, meta-memory in dyslexics and normal control as a function of the category question**.

**Group**	**Memory**															
	**Prospective memory**								**Retrospective memory**							
	**Short-term**				**Long-term**				**Short-term**				**Long-term**			
	**Self-cued**		**Envt-cued**		**Self-cued**		**Envt-cued**		**Self-cued**		**Envt-cued**		**Self-cued**		**Envt-cued**	
	**LM**	**HM**	**LM**	**HM**	**LM**	**HM**	**LM**	**HM**	**LM**	**HM**	**LM**	**HM**	**LM**	**HM**	**LM**	**HM**
Dyslexia	3.03 (0.80)	2.21 (0.64)	2.44 (0.72)	2.12 (0.70)	2.59 (1.01)	1.71 (0.53)	2.26 (0.78)	1.50 (0.43)	2.89 (0.98)	2.15 (0.92)	2.01 (0.81)	1.76 (0.40)	2.26 (0.91)	1.62 (0.49)	2.28 (1.00)	1.88 (0.67)
Normal Control	2.70 (0.79)	2.14 (0.57)	2.48 (0.91)	1.75 (0.59)	1.96 (0.63)	1.79 (0.63)	2.04 (0.78)	1.62 (0.72)	2.48 (0.70)	1.92 (0.73)	1.79 (0.76)	1.50 (0.52)	2.14 (0.74)	1.49 (0.47)	2.36 (0.69)	1.60 (0.66)
Total	2.88 (0.80)	2.16 (0.59)	2.46 (0.80)	1.87 (0.64)	2.31 (0.91)	1.76 (0.59)	2.16 (0.78)	1.58 (0.64)	2.71 (0.88)	1.99 (0.72)	1.91 (0.79)	1.58 (0.50)	2.19 (0.83)	1.53 (0.47)	2.31 (0.87)	1.69 (0.67)

*LM, Low meta-memory; HM, High meta-memory; Envt-cued, Environmental cue*.

Analysis of variance revealed that all the main effects were significant except cue as shown in Table 2. Memory (Prospective vs. Retrospective): [*F*_(1, 111)_ = 64.70 *p* < 0.001, η^2^ = 0.37], Term (short vs. Long) [*F*_(1, 111)_ = 8.87 *p* < 0.001, η^2^ = 0.0.074], Group (Developmental dyslexia vs. Control Group) [*F*_(1, 111)_ = 6.51 *p* < 0.01, η^2^ = 0.06], [*F*_(1, 111)_ = 60.72 *p* < 0.001, η^2^ = 0.0.35].

**Table 2:** **Summary of analysis of variance of performance scores on group (Dyslexi vs. Normal control), Meta-memory (Low vs. High), Memory (Prospective vs. Retrosective), Term (Short vs. Long), and Cue (Self vs. Environment)**.

**Source**	**SS**	***df***	**Mean square**	***F***	**Sig**.	**Partial eta squared**
**BETWEEN SUBJECTS**						
Group	7.05	1	7.05	6.51	0.01	0.06
Meta-memory	65.77	1	65.77	60.72	0.001	0.35
Error	120.23	111	1.08			
**WITHIN SUBJECTS**						
Memory	33.80	1	33.80	64.70	0.001	0.37
Error	58.12	111	0.52			
Term	4.16	1	4.16	8.87	0.01	0.07
Error	52.02	111	0.47			
Cue × Term	10.88	1	10.88	30.77	0.001	0.22
Cue × Term × Group × Meta-memory	2.00	1	2.00	5.67	0.01	0.05
Error	39.22	111	0.35			
Memory × Cue × Term	2.06	1	2.06	4.58	0.03	0.04
Error (Memory × Cue × Term)	49.82	111	0.45			

The analyses revealed that there was a group difference in all the components of prospective and retrospective memory. The means and standard deviations of memory components were: performance of dyslexic participants on prospective memory, short term and self cued (Mean = 2.75, *SD* = 0.84), short term and environmental cued (*M* = 2.33, *SD* = 0.92), long term and self cued (*M* = 2.29, *SD* = 0.97) and long term and environmental cued (*M* = 2.01, *SD* = 0.77). Whereas performance of dyslexics population on retrosective memory was: retrospective memory, short term and self cued (*M* = 2.65, *SD* = 0.96), short term, enviromental cued (*M* = 1.93, *SD* = 0.71), long term self cued (*M* = 2.02, *SD* = 0.84), long term and environemtal cued (*M* = 2.01, *SD* = 0.77). Similarly, performaces of normal control were: prospective memory, short term and self cued (Mean = 2.38, *SD* = 0.72), short term and environmental cued (*M* = 2.07, *SD* = 0.83), long term and self cued (*M* = 1.87, *SD* = 0.63) and long term and environmental cued (*M* = 1.80, *SD* = 0.77). Whereas performance of normal control population on retrospective memory was: retrospective memory, short term and self cued (*M* = 2.16, *SD* = 0.77), short term, enviromental cued (*M* = 1.63, *SD* = 0.65), long term self cued (*M* = 1.77, *SD* = 0.68), long term and environemtal cued (*M* = 1.93, *SD* = 0.77). Dyslexics population with metamemory performace better than low metamemroy dyslexics. A significant interaction was obtained between term and cue [*F*_(1, 111)_ = 30.77, *p* < 0.001, η^2^ = 0.22]. Further, the interactions memory × term × cue [*F*_(1, 111)_ = 4.58, *p* < 0.03, η^2^ = 0.04] and cue × term × metamemory × group [*F*_(1, 111)_ = 5.67, *p* < 0.001, η^2^ = 0.05] were also significant. However, no other interaction was significant.

**Figure 1:**
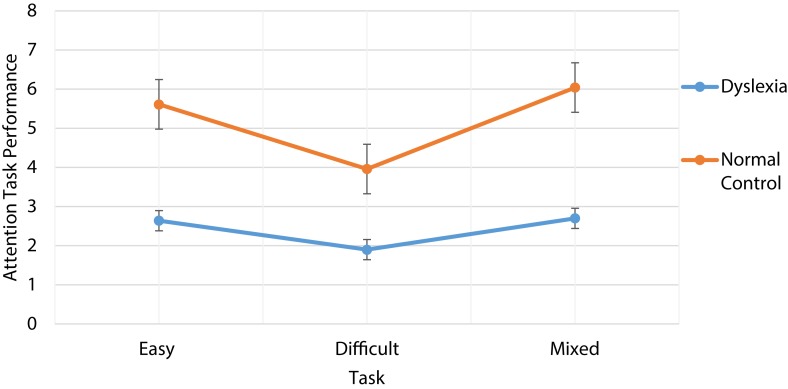
**Performance of dyslexic and normal control of easy difficult and mixed attention task**.

Second, analysis of variance (ANOVA) was conducted to see the effect of attention on group (developmental dyslexic vs. control group). The analysis employed 3 (Attention: easy vs. difficult vs. mixed task) × 2 (Group: Dyslexic vs. Normal control) mixed factorial design. The first factor was within design. The mean and standard deviation of group and attention was shown in Figure 1.

The analysis revealed that both main effects and interaction were significant, group (dyslexia vs. normal control): [*F*_(1, 113)_ = 15.24 *p* < 0.001, η^2^ = 0.12], and attention: [*F*_(2, 226)_ = 6.58 *p* < 0.002, η^2^ = 0.06]. The interaction between attention and group [*F*_(1, 111)_ = 64.70 *p* < 0.001, η^2^ = 0.37]was also significant as shown in Table 3.

**Table 3:** **Summary of analysis of variance of performance scores on group (Dyslexia vs. Normal control), and attention (Easy vs. Difficult vs. Mixed)**.

**Source**	**SS**	***df***	**Mean square**	***F***	**Sig**.	**Partial eta squared**
**BETWEEN SUBJECTS**						
Group	12.01	1	12.01	15.23	0.001	0.12
Error	89.07	113	0.79			
**WITHIN SUBJECTS**						
Attention	68.84	2	34.42	130.66	0.001	0.54
Attention × Group	3.47	2	1.73	6.85	0.002	0.06

## Discussion

The aim of the present study was to investigate prospective and retrospective memory performance among children with and without developmental dyslexia. There is clear result that normal control outperforms dyslexic children on prospective and retrospective memory tasks. There was deterioration in prospective memory performance as compared to retrospective memory task. This was expected because prospective memory being high on self-initiated process, it requires more cognitive resources to perform a given task. Developmental dyslexia is a disorder of neurocognitive dysfunction, as a result of that dyslexic children performance on prospective memory task will worse than performance on retrospective memory. The result can be explained in the light of phonological and visuo-attentional deterioration among dyslexic participants. Dyslexic children find it difficult to pay attention to words presented to them. Similarly, working memory plays significant role both in prospective memory performance and dyslexia. As a result there is more pronounced performance deterioration among dyslexic than normal control.

Further, there was a deterioration of performance of each component of prospective memory among dyslexic children as compared to normal control group. There was deterioration among dyslexic performance on short term prospective memory task (*M* = 2.45) as compared to short term retrospective memory task (*M* = 2.20). However, there was no difference on performance on long term prospective memory (*M* = 2.01) and long term retrospective memory in dyslexic children. Short-term self-cued errors were rated higher than long-term and environmentally-cued for both the memories. This can be explained as retrieval failure or “momentary lapses of intention” (Craik and Kerr, 1996; West and Craik, 1999) in the case of prospective memory. The probably explanation of this finding could be that realization of intention in distant future may not require self-initiated process which may not put extra burden on cognitive resource among dyslexic children. This result is in accord with previous findings (Khan and Sharma, 2007). Results revealed that performance on self–cued was more difficult to realize as compared to under environmentally-cued for dyslexic group. Again, the result is as expected. Realization of delayed intention is easy if there is cue in the environment which may prompt an individual to start performing the task as compared to when there is nothing in the environment to provide a cue for the realization of intention. Self-cued performance requires constant monitoring for the task to be performed consequently it depletes cognitive resource of an individual (Khan et al., 2008; Altgassen et al., 2010). As far as performance on prospective and retrospective memory of dyslexic and normal control is concerned, there was significant difference between dyslexic (*M* = 2.17) and normal control (*M* = 2.00).

Meta-memory influences both dyslexic and normal control on prospective and retrospective memory. However, meta-memory affected dyslexic much more than normal control group. The possible reason might be that prospective memory requires greater self-initiation than retrospective memory. Since people who are good at meta-memory require less external aid to perform memory task successfully, this might lead to a direct relation between meta-memory and prospective memory. Similarly, there was pronounced difference in the performance of prospective and retrospective memory among dyslexic population.

One of the potent and plausible hypothesis states that phonological deficit of verbal working memory is one of the cause of the developmental dyslexia. Poor phonological processing is considered to be related to poor reading ability. Weak phonological coding is instrumental in the development of developmental dyslexia. Further, working memory impairment is associated with weak phonological coding (Vellutino et al., 2004). Therefore, it can be safely stated that impaired working memory facilitates weak phonological coding, and consequently deficit in accessing phonological representation. However, recently it has been observed that visual-spatial attention is potential cause of developmental dyslexia (Vidyasagar and Pammer, 1999). The current study focused on self- report of attention among dyslexic and normal control group. Attentional task was divided into simple, difficult and mixed task (both simple and difficult). The results were in accord with expected lines. Simple attentional task (*M* = 2.64) was easier than difficult task (*M* = 1.90). However, mixed attentional task was easiest one to perform among both dyslexic and normal control group. Similarly, normal control group performance was better than dyslexics group across simple attentional, difficult and mixed tasks. The probable explanation for the results is that dyslexics find difficult to focus their attention on a word. As a result, their performance deteriorates across cognitive domains especially in attentional and memory tasks.

The present study provides only perceived memory and attention rather than actual performance. Investigation of the above questions on the basis of experimental studies can provide further insight into the role of attention, meta-memory and prospective and retrospective memory performance among dyslexic population.

### Conflict of interest statement

The author declares that the research was conducted in the absence of any commercial or financial relationships that could be construed as a potential conflict of interest.
